# Suppressors of Cytokine Signaling (SOCS)1 and SOCS3 Proteins Are Mediators of Interleukin-10 Modulation of Inflammatory Responses Induced by *Chlamydia muridarum* and Its Major Outer Membrane Protein (MOMP) in Mouse J774 Macrophages

**DOI:** 10.1155/2020/7461742

**Published:** 2020-06-24

**Authors:** Skyla A. Duncan, Rajnish Sahu, Saurabh Dixit, Shree R. Singh, Vida A. Dennis

**Affiliations:** Center for NanoBiotechnology Research (CNBR), Department of Biological Sciences, Alabama State University, 1627 Harris Way, Montgomery, AL 36104, USA

## Abstract

The immunopathology of chlamydial diseases is exacerbated by a broad-spectrum of inflammatory mediators, which we reported are inhibited by IL-10 in macrophages. However, the chlamydial protein moiety that induces the inflammatory mediators and the mechanisms by which IL-10 inhibits them are unknown. We hypothesized that *Chlamydia* major outer membrane protein (MOMP) mediates its disease pathogenesis, and the suppressor of cytokine signaling (SOCS)1 and SOCS3 proteins are mediators of the IL-10 inhibitory actions. Our hypothesis was tested by exposing mouse J774 macrophages to chlamydial stimulants (live *Chlamydia muridarum* and MOMP) with and without IL-10. MOMP significantly induced several inflammatory mediators (IL-6, IL-12p40, CCL5, CXCL10), which were dose-dependently inhibited by IL-10. Chlamydial stimulants induced the mRNA gene transcripts and protein expression of SOCS1 and SOCS3, with more SOCS3 expression. Notably, IL-10 reciprocally regulated their expression by reducing SOCS1 and increasing SOCS3. Specific inhibitions of MAPK pathways revealed that p38, JNK, and MEK1/2 are required for inducing inflammatory mediators as well as SOCS1 and SOCS3. Chlamydial stimulants triggered an M1 pro-inflammatory phenotype evidently by an enhanced nos2 (M1 marker) expression, which was skewed by IL-10 towards a more M2 anti-inflammatory phenotype by the increased expression of mrc1 and arg1 (M2 markers) and the reduced SOCS1/SOCS3 ratios. Neutralization of endogenously produced IL-10 augmented the secretion of inflammatory mediators, reduced SOCS3 expression, and skewed the chlamydial M1 to an M2 phenotype. Inhibition of proteasome degradation increased TNF but decreased IL-10, CCL5, and CXCL10 secretion by suppressing SOCS1 and SOCS3 expressions and dysregulating their STAT1 and STAT3 transcription factors. Our data show that SOCS1 and SOCS3 are regulators of IL-10 inhibitory actions, and underscore SOCS proteins as therapeutic targets for IL-10 control of inflammation for *Chlamydia* and other bacterial inflammatory diseases.

## 1. Introduction

*Chlamydia* is one of the most prevalent bacterial sexually transmitted infections (STIs) worldwide, with an estimated annual incidence of 1.7 million cases in the United States [1]. The pathogenic agent responsible for this infection is *Chlamydia trachomatis*, a gram-negative and intracellular anaerobe bacterium [2] that causes mucosal infections of the genital, anorectal, and oropharyngeal surfaces in humans [3]. Genital *Chlamydia* is associated with significant reproductive morbidity, including tubal factor infertility, with women being more disproportionately affected than men [4, 5].

The unique developmental cycle of *C. trachomatis* allows for its intracellular reproduction while infecting neighboring cells, resulting in persistent disease or re-infection even after treatment [3, 6, 7]. *C. trachomatis* also possesses diverse virulence factors including, its major outer membrane protein (MOMP) that exhibits high immunogenic potential [8, 9]. We have published that MOMP or its peptide derivative encapsulated within biodegradable polymeric nanoparticles such as chitosan [10], PLGA [poly (D, L-lactide-co-glycolide)] [11] and PLA-PEG [poly(lactic acid)-poly(ethylene glycol)] [12], hold promise as *Chlamydia* nanovaccine candidates since they potentiated and enhanced adaptive immune responses. Notwithstanding, other researchers have reported that *C. trachomatis* infection induces overproduction of a variety of inflammatory cytokines (Interleukin-6 (IL-6), Interleukin-8 (IL-8), Interleukin-12p40 (IL-12p40)), Tumor necrosis factor (TNF), Growth-regulated oncogene-alpha (GRO*α*), Granulocyte-macrophage colony-stimulating factor (GM-CSF)) [13–16] and chemokines (Chemokine (C-C motif) ligand 5 (CCL5), Chemokine interferon-*γ* inducible protein 10 kDa (CXCL10)) [15, 17, 18], which are implicated in chlamydial immunopathology [6, 19]. During acute infection, *C. trachomatis* induces these inflammatory mediators to diminish the host immune response [20], and as the infection prolongs or repeated infections occur, more inflammatory mediators and immune cells are released to combat the infection [21, 22]. Excessive production of inflammatory mediators contributes significantly to the disease manifestation by damaging neighboring cells [23, 24]. Such observations confirm an intimate relationship between *Chlamydia* and the host immune system. In particular, these studies suggest that the ability of *Chlamydia* to hijack the immune response can account for some of the complicated pathologies associated with chlamydial diseases.

On the other hand, despite having a low mortality rate, *C. trachomatis* causes serious [22] complications that result in irreversible damage to the infected population if left untreated, thus becoming a considerable burden among high-risk people [2]. The incidence of *C. trachomatis* infections continues to rise despite over two decades of national screening efforts in the United States. Notably, antibiotics against *Chlamydia* is very effective; however, due to the asymptomatic nature of the disease, antibiotic treatment is inadequate in an already established and persistent infection [25]. Consequently, the development of an effective treatment would be invaluable for reducing the worldwide incidence and prevalence of *C. trachomatis* infections. To this end, various studies exploring the potential use of molecules with immune therapeutic properties [19, 26, 27] are generating increasing interest, particularly for developing new methods to curtail the devastating consequences of the inflammatory aspect of the disease.

Interleukin-10 (IL-10) is a molecule with potent anti-inflammatory therapeutic properties [28–30]. It is a multi-functional immuno-regulatory cytokine that plays a central role in suppressing inflammation, preventing damage to the host, and maintaining normal tissue homeostasis [31, 32]. IL-10 has significant effects on immune cells, specifically related to antigen presentation, the release of immune mediators, and phagocytosis [32, 33]. The immunosuppressive activity of IL-10 is mediated by its heterodimeric IL-10 receptor (IL-10R1, IL-10R2), whose ligation activates the Janus kinases (JAKs), signal transducer and activator of transcription proteins (STATs) (JAK/STAT) signaling and subsequently leading to massive changes in the expression profile of immuno-modulatory genes [34]. These genes effectively serve to enhance the IL-10 inhibitory, tolerance, and scavenger functions of monocytes and macrophages [35].

IL-10 and other cytokines that are involved in the regulation of the immune system and inflammation [36] use the JAK/STAT pathway, which in turn is regulated, especially by the suppressors of cytokine signaling (SOCS) proteins [34]. SOCS proteins fine-tune immune responses by binding to JAK and other cytokine receptors to suppress signaling events, thereby serving as key physiological regulators of inflammation [37, 38]. Reportedly, SOCS1 and SOCS3 are essential regulators of adaptive immunity, making them ideal therapeutic targets for inflammatory diseases such as *Chlamydia*. More importantly, IL-10 induces the expression of SOCS1 and SOCS3 in macrophages, suggesting that SOCS proteins may be mediators of its anti-inflammatory actions [39].

We previously reported that infection of mouse J774 macrophages with live *C. trachomatis* induces the release of IL-6, TNF, and IL-8, which were inhibited by IL-10 [27]. However, the chlamydial protein moiety responsible for inducing these inflammatory mediators and the mechanism(s) by which IL-10 inhibits them in macrophages are still unknown. We hypothesized that the *Chlamydia* MOMP mediates its disease pathogenesis, and SOCS1 and SOCS3 proteins are mediators of the IL-10 modulatory actions in macrophages. Our hypothesis was tested by first exposing J774 macrophages to dose-dependent concentrations of chlamydial stimulants [live *C. muridarim* (Cm) and its recombinant MOMP] with and without exogenously added IL-10 to decipher the primary inducer of inflammatory mediators that are inhibited by IL-10. Second, we evaluated the time- and dose-dependency effect of stimulants alone or combined with IL-10 on the mRNA gene transcripts and protein expression of SOCS1 and SOCS3. Third, specific inhibitions of mitogen-activated protein kinase (MAPK) pathways (p38, JNK, and MEK1/2) were conducted to elucidate their requirements for the induction of inflammatory mediators as well as SOCS1 and SOCS3. Fourth, the role of endogenously produced IL-10 in regulating inflammatory mediators, macrophage phenotypes, and SOCS1 and SOCS3 expressions was investigated. Fifth, we examined the effect of IL-10 polarization on M1 and M2 macrophage phenotypes and the expression of SOCS1 and SOCS3 for its modulatory actions. Lastly, proteasome inhibition was explored to ascribe a functional role for SOCS1 and SOCS3 as mediators of the IL-10 anti-inflammatory effect in macrophages. We present our findings and discuss the multifaceted mechanisms by which IL-10 controls chlamydial inflammatory responses in macrophages.

## 2. Materials and Methods

### 2.1. Cell Line

Mouse J774 macrophages were obtained from the American Type Culture Collection (ATCC, Manassas, VA, USA) and cultured in Dulbecco Modified Eagle Medium (DMEM) (ATCC) supplemented with 10% heat-inactivated fetal bovine serum (FBS) (Gibco, Grand Island, NY, USA) and 1 *μ*g/mL antibiotic and antimycotic (Gibco) complete medium [40]. Cells were maintained at 37°C in a humidified incubator containing 5% CO_2_ for various time-periods, depending on the experimental procedure.

### 2.2. *Chlamydia* Stimulants

Cm [strain Nigg II; previously called *C. trachomatis* mouse pneumonitis (MoPn) biovar] expressed as inclusion forming units (IFU/mL) was purchased from Virusys Corporation (Taneytown, MD, USA) [41]. The purified live Cm elementary bodies (EBs) were suspended in Sucrose-Phosphate Glutamic acid (SPG) buffer and stored in small aliquots at -80°C until used. Cm was incubated at a multiplicity of infection (MOI) of 0.5, 1, and 2 with macrophages in antibiotic-free DMEM supplemented with 10% FBS. The recombinant major outer membrane protein (rMOMP) was cloned, as previously reported [11] and incubated with macrophages in complete media at concentrations ranging from 0.1, 1, and 10 *μ*g/mL.

### 2.3. Stimulation of Macrophages

Several experimental studies were considered to determine the effect of mouse recombinant IL-10 (BD Biosciences, San Jose, CA, USA) and chlamydial stimulants on the expression of cytokines and chemokines as well as SOCS1 and SOCS3.

Macrophages (1 × 10^6^/mL) were incubated in 12-well plates and exposed to dose-dependent additions of rMOMP (0.1, 1 and 10 *μ*g/mL) and Cm (MOI of 0.5, 1 and 2) in the presence or absence of IL-10 (10 ng/mL) for 24 h. For the IL-10-dose-dependent study, macrophages (1 × 10^6^/mL) were incubated in 12-well plates with various concentrations of IL-10 (0.1, 1 and 10 ng/mL) in the presence or absence of rMOMP (10 *μ*g/mL) and Cm (MOI of 2) for 24 h. Time-kinetics studies were conducted by incubating macrophages (1 × 10^6^/mL) in 24-well plates with various concentrations of IL-10 (0.1, 1 and 10 ng/mL) in the presence or absence of rMOMP (10 *μ*g/mL) for 0.5, 1, 2 and 24 h post-stimulation.

Pathway inhibition studies were performed using pharmacological inhibitors for p38 MAPK, JNK, and MEK1/2 signaling pathways. Macrophages (1 × 10^6^ cells/mL) were pre-incubated with 20 *μ*M pathway-specific inhibitor: SB203350 (p38 MAPK), SP600125 (JNK) and U0126 (MEK1/2) all from EMD Millipore Corporation (Billerica, MA, USA). After 1 h of pre-incubation, cells were exposed to rMOMP (10 *μ*g/mL) in the presence and absence of IL-10 (10 ng/mL) for an additional 24 h. The 20 *μ*M concentration and 24 h inhibition time-point used for all inhibitors were optimal conditions as predetermined in our laboratory [19].

For the exogenous study, recombinant IL-10, IL-6, and TNF (BD Biosciences) each at 10 ng/mL was added to macrophages in the presence and absence of rMOMP (10 *μ*g/mL) and Cm (MOI of 2). For the endogenous study, neutralization of endogenously produced IL-10, IL-6, and TNF was performed by pre-incubating macrophages with a neutralizing rat anti-mouse IL-10 antibody (Ab), anti-mouse IL-6 Ab and anti-mouse TNF Ab (each at 25 *μ*g/mL) Normal rat IgG1 Ab (25 *μ*g/mL) served as the isotype control. After 30 min of pre-incubation at 37°C, cells were exposed to rMOMP (10 *μ*g/mL) or Cm (MOI of 2) for an additional 24 h.

M1 and M2 macrophage phenotypes were determined using stimulated macrophages from the dose-dependent and neutralization studies above. For M1 and M2 polarization studies, macrophages were pre-incubated with Interferon-gamma (IFN-*γ*), Interleukin-4 (IL-4), Interleukin-13 (IL-13) or IL-10 (each at 10 ng/mL) for 1 h before stimulation with rMOMP (10 *μ*g/mL) in the presence and absence of IL-10 (10 ng/mL) for an additional 24 h.

For inhibition of proteasome degradation, macrophages (1 × 10^6^/mL) were pre-treated with the FDA approved proteasomal inhibitor; Bortezomib (Btzb) (Millipore-Sigma Aldrich, St. Louis, MO, USA) at concentrations of 1 and 20 nM for 1 h, followed by stimulation with rMOMP (1 *μ*g/mL) and Cm (MOI of 2) with or without added IL-10 (10 ng/mL) for an additional 24 h.

All stimulated macrophage cultures were incubated at 37°C under 5% CO_2_ for various time-points ranging between 30 min to 24 h depending on the specific experiment. Post-stimulation, cell-free supernatants were collected by centrifugation at 450 × g for 10 min at 4°C and stored at -80°C until used for cytokine and chemokine ELISAs. Cell pellets were either used for RNA extraction or flow cytometry analysis, as described below.

### 2.4. Quantification of Cytokines and Chemokines

For all studies, cytokines and chemokines were quantified in cell-free supernatants using cytokine and chemokine specific ELISAs as reported [11, 40]. Cytokine kits (IL-6, IL-10, IL-12 p40, TNF) were purchased from BD Biosciences and BioLegend (San Diego, CA, USA). Chemokine kits (CCL5 and CXCL10) were purchased from R&D Systems (Minneapolis, MN, USA). Absorbance was read at 450 nm using a microplate reader (Hidex Chameleon, IN, USA). The detection limits were 4 pg/mL (IL-6, IL-10, IL-12p40 and TNF), 31 pg/mL (CCL5), and 62 pg/mL (CXCL10). All ELISAs were run in triplicates and repeated at least 4 times.

### 2.5. RNA Extraction and Quantitative Real Time-PCR (qRT-PCR)

Total RNA was isolated from unstimulated and stimulated cells using Qiagen RNeasy mini plus Kit (Qiagen Inc., Valencia, CA, USA), which included a DNase-I digestion step or the use of gDNA eliminator columns. The resulting RNA samples were transcribed into complementary deoxyribonucleic acid (cDNA) using the High Capacity cDNA Reverse Transciption Kit (Applied Biosystems, Foster City, CA, USA). Next, TaqMan® qRT-PCR was employed as described [42] to assess the messenger ribonucleic acid (mRNA) gene transcripts of the following genes (socs1 [Mm00782550_s1], socs3 [Mm00545913_s1], stat1 [Mm01219775_m1], stat3 [Mm01219775_m1], macrophage mannose receptor (mrc1) [Mm01329362_m1], arginase 1 (arg1) [Mm00475988_m1] and nitric oxide synthase 2 (nos2) [Mm00440502_m1]) using TaqMan® gene expression assays (Applied Biosystems) as reported [19, 26, 41]. Amplification of gene transcripts was performed according to the manufacturer's protocol using ABI ViiA™ 7 real-time PCR (Applied Biosystems) and standard amplification conditions. The relative changes in gene expression were calculated using the equation: ^2−^*ΔΔ*CT where all values were normalized with respect to the “housekeeping” gene glyceraldehyde 3-phosphate dehydrogenase (GAPDH) [Mm99999915_g1] mRNA levels. Amplification using 50 ng RNA was performed in a total volume of 20 *μ*L. Each real-time PCR assay was performed in triplicates and repeated at least 4 times.

### 2.6. Immunofluorescence Microscopy

Macrophages (2.5 × 10^4^ cells/well) were cultured on sterilized 8-well chamber slides and exposed to Cm (MOI of 1) or rMOMP (1 *μ*g/mL) in the presence or absence of IL-10 (10 ng/mL) as described [27]. After 24 h post-exposure, the supernatants were removed; the cells were washed with phosphate-buffered saline (PBS), fixed with 2% paraformaldehyde (PFA), and then permeabilized in permeabilization buffer (PB) containing Saponin (0.5%) for 0.5 h. Permeabilized cells were subsequently subjected to immunostaining using fluorochrome-conjugated anti-SOCS3 Alexa Fluor® 647 primary antibody (Santa Cruz Biotechnology, Dallas, TX, USA) diluted in PB. After 1 h incubation at room temperature (RT), the cells were washed with PBS then stained with DAPI and mounted using Vectashield® Hardset™ anti-fade mounting medium with Phalloidin (Vector Laboratories, Burlingame, CA, USA). The slides were then visualized under an epifluorescence microscope equipped with Digital sight DS-Qi1 High-Definition camera and NIS-Elements AR software (Nikon Instrument, Melville, NY, USA).

### 2.7. Flow Cytometry

Macrophages were stimulated (section 2.3 above), washed and blocked with Fc blocking Ab (BD Biosciences) in fluorescent-activated cell sorting (FACS) buffer (PBS, 0.1% NaN_3_, 1.0% FBS for 15 min at 4°C [11, 12]. The cells were washed and stained with fluorochrome-conjugated antibodies (Abs) (SOCS1-Alexa Fluor® 488, SOCS3-Alexa Fluor® 647, NOS2-PE and MRC1/CD206-Alexa Fluor® 680 (Santa Cruz Biotechnology, Dallas, TX, USA)) for 30 min at 4°C, and then washed, fixed with 2% paraformaldehyde solution (PFA) for 20 min at 4°C. Data were acquired on a BD FACS Canto II flow cytometer (BD Bioscience) with at least 1 × 10^5^ events for each sample and analyzed using FCS Express 6 FLOW (De Novo Software, Pasadena, CA, USA).

### 2.8. Statistics Analysis

Data are expressed as the mean ± standard deviation (SD) of samples run in triplicates, and each experiment was repeated at least 3 to 4 different times. Statistical analyses were performed using one- or two-way analysis of variance (ANOVA) followed by Tukey's Post-test using GraphPad Prism 6 Software (GraphPad Software, Inc., San Diego, CA, USA). Statistical significance was established and P values <0.05 were considered as statistically significant (∗*P* < 0.05; ∗∗*P* < 0.01, ∗∗∗*P* < 0.001 and ∗∗∗∗*P* < 0.0001).

## 3. Results

### 3.1. *Chlamydia* MOMP Induces Copious Levels of Cytokines and Chemokines That Are Dose-Dependently Inhibited by Exogenous IL-10 in Macrophages

**Figure 1:**
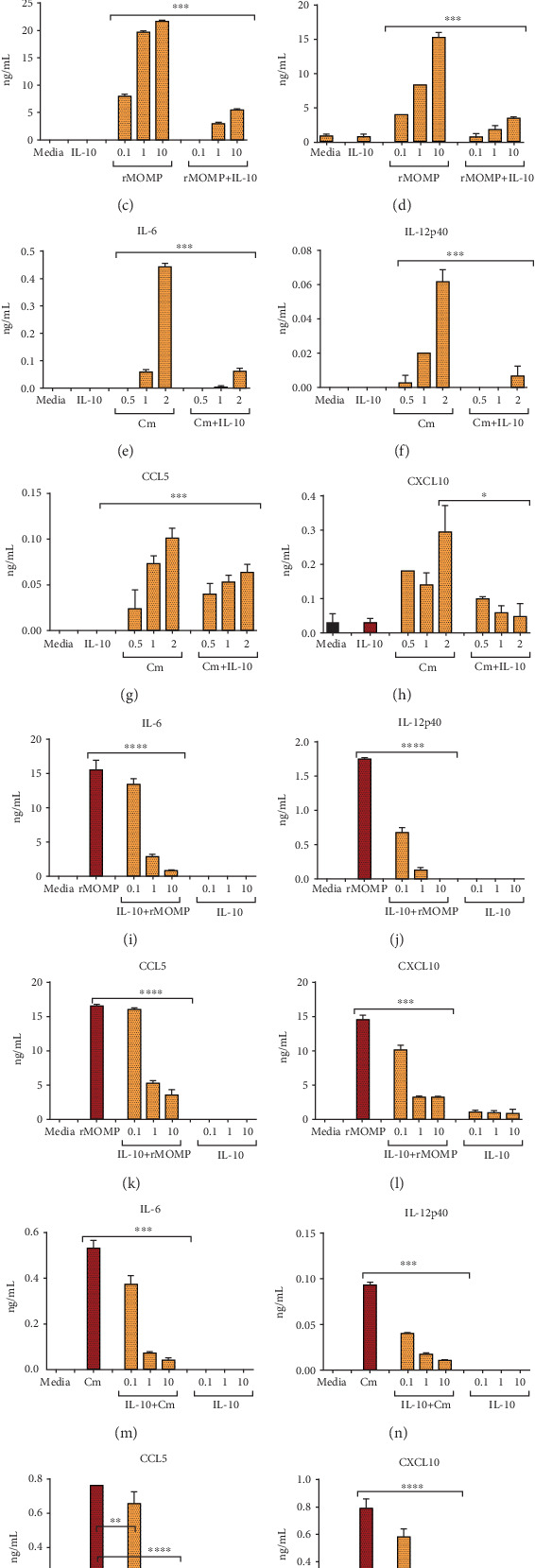
MOMP is a potent inducer of inflammatory mediators that are dose-dependently inhibited by added IL-10 to macrophages. For chlamydial stimulant dose-dependent studies, macrophages (10^6^ cells/mL) were stimulated with rMOMP at 0.1, 1 and 10 *μ*g/mL (A-D) or live Cm at MOIs of 0.5, 1 and 2 (E-H) in the presence and absence of IL-10 (10 ng/mL). For IL-10 dose-dependent studies, macrophages were stimulated with rMOMP at 10 *μ*g/mL (I-L) or Cm at an MOI of 2 (M-P) in the presence and absence of varying dosages of IL-10 (0.1, 1 and 10 ng/mL). Cell-free culture supernatants were collected after 24 h to quantify cytokines and chemokines by specific ELISAs. Asterisks indicate significant differences between stimulated macrophages alone and those with added IL-10 (*P* <0.05). *P* values were calculated by the use of ANOVA followed by Turkey's Post-test using GraphPad Prism 6 Software. Each bar represents the mean ± SD of samples run in triplicates. Each experiment was repeated at least 4 times with similar results and shown is a representative experiment.

The secretion of inflammatory cytokines and chemokines plays an integral role in the pathogenesis of chlamydial diseases. We previously established that *Chlamydia* induces the secretion of several inflammatory cytokines and chemokines by mouse macrophages [19, 26, 27] and that IL-10 effectively inhibited the production of TNF, IL-6, and IL-8 as elicited by *Chlamydia* in mouse macrophages [27]. Because our previous study employed live *Chlamydia* to test the inhibitory effect of IL-10 on secreted cytokines, and knowingly that live bacteria are composed of a plethora of stimulatory molecules, we deemed it necessary to identify a protein moiety that is the major stimulator of inflammatory responses in macrophages. To this end, MOMP was selected because it is the most dominant and immunogenic surface protein on *Chlamydia* [43–46]. Macrophages were exposed to rMOMP (0.1, 1 and 10 *μ*g/mL) or Cm (MOIs of 0.5, 1 and 2) with and without added IL-10 (10 ng/mL). All concentrations of rMOMP significantly (*P* <0.001) elicited the production of cytokines and chemokines in a concentration-dependent manner, except IL-12p40. Of interest, IL-10 inhibited their release and was highly effective at lower stimulant concentrations, suggesting IL-10 inhibitory action is dependent on the concentration of mediators in the milieu (Figures 1(a)–1(d)). Cm stimulated several-fold less cytokines and chemokines, and the addition of IL-10 significantly (*P* <0.001 to 0.05) reduced their levels (Figures 1(e)–1(h)). However, an MOI of 2 corresponds to approximately 0.2 *μ*g/mL, which may help explain the lower stimulatory potential of Cm.

To assess whether the IL-10 anti-inflammatory effect is concentration-dependent, we evaluated a dose-dependent inhibitory effect of IL-10 on the expression of inflammatory mediators in chlamydial-stimulated macrophages. Neither IL-10 or unstimulated macrophages induced cytokines or chemokines. As expected, IL-10 at all tested concentrations (0.1, 1, and 10 ng/mL) significantly (*P* <0.001 to 0.0001) inhibited cytokines and chemokines in a dose-dependent fashion, being more effective at the 10 ng/mL concentration (Figures 1(i)–1(p)). Notably, IL-10 was more efficient in inhibiting cytokines than chemokines. Our results confirm that MOMP is a significant inducer of inflammatory mediators in macrophages and that the IL-10 maximal inhibitory effect is dependent on the concentration of mediators in the milieu. Notably, the data also shed new light on the IL-10-mediated inhibition of both chlamydial-induced cytokines and chemokines.

### 3.2. SOCS1 and SOCS3 Are Differentially Induced at the Transcriptional Level and Regulated by Exogenous IL-10 in *Chlamydia*-Stimulated Macrophages

**Figure 2:**
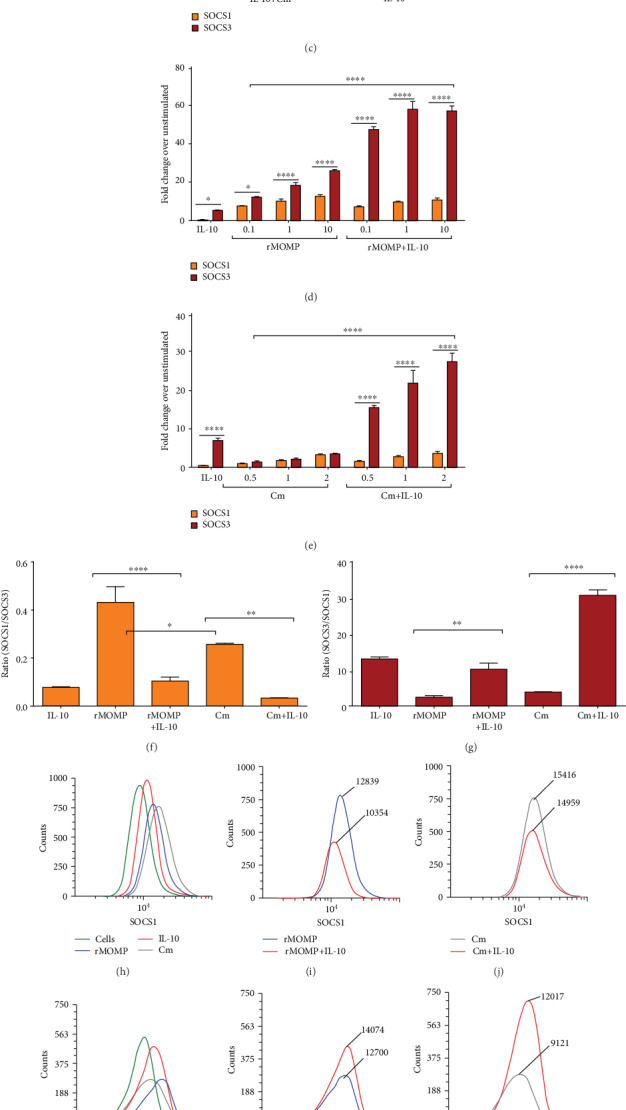
SOCS1 and SOCS3 transcriptional and translational expressions are differentially induced and regulated by chlamydial stimulants and exogenous IL-10 in macrophages. Macrophages were exposed to dose-dependent concentrations of IL-10 (0.1, 1 and 10 ng/mL) in the presence and absence of rMOMP (10 *μ*g/mL). RNA samples were collected at 0, 0.5, 1, 2, and 24 h post-stimulation to quantify the mRNA gene transcript of SOCS1 (A) and SOCS3 (B). RNA samples were collected at 24 h from macrophages stimulated with Cm (MOI of 2) with and without dose-dependent concentrations of IL-10 to quantify SOCS1 and SOCS3 transcripts (C). Macrophages were stimulated with rMOMP (0.1, 1 and 10 *μ*g/mL) or Cm (MOI of 0.5, 1 and 2) with and without IL-10 (10 ng/mL) and SOCS1 and SOCS3 transcripts were quantified at 24 h post-stimulation (D-E). For TaqMan qRT-PCR, all values were normalized with respect to the mRNA levels of the “housekeeping” gene that codes for GAPDH. Results are presented as fold increase over the control (i.e., the level in unstimulated cells. Calculations of SOCS1/SOCS3 (F) and SOCS3/SOCS1 (G) ratios in macrophages exposed to rMOMP (10 *μ*g/mL) or Cm (MOI of 2) in the presence and absence of IL-10 (10 ng/mL). Macrophages were exposed to rMOMP (1 *μ*g/mL) or Cm (MOI of 2) in the presence and absence of IL-10 to evaluate SOCS1 and SOCS3 expression by flow cytometry (H-M) and SOCS3 by immunofluorescence microscopy (N) 24 h post-incubation. An asterisk indicates significant differences (*P* <0.05), and *P* values were calculated as described in Figure 1 legend. Each bar represents the mean ± SD of samples run in triplicates, and each experiment was repeated at least 4 times with a representative experiment shown.

It has been previously shown that SOCS1 and SOCS3 expressions can be induced in macrophages by IL-10 [39, 47–52], and their expressions are frequently increased in many inflammatory diseases [37, 53–56], such as *Chlamydia* [57, 58]. Therefore, we aimed to decipher the molecular mechanism(s) by which IL-10 inhibits *Chlamydia* inflammatory responses in macrophages by focusing on SOCS1 and SOCS3. Both dose-dependent and time-kinetics experiments were performed using macrophages stimulated with rMOMP (10 *μ*g/mL) in the presence and absence of IL-10 (0.1, 1 and 10 ng/mL) for various time-points (0.5, 1, 2 and 24 h). The time-kinetics and dose-dependent studies revealed that IL-10 marginally induced SOCS1 in contrast to SOCS3 mRNA gene transcripts, particularly at the 10 ng/mL concentration (Figures 2(a) and 2(b)). IL-10 rapidly induced SOCS3 in macrophages as early as 0.5 h, with a steady increase up to 24 h. We observed that rMOMP induced marked SOCS3 in contrast to SOCS1 expression over the 24 h period, suggesting their differential stimulatory expressions. Co-incubation of IL-10 and rMOMP confirmed the differential regulation of SOCS1 and SOCS3 by IL-10 as early as 0.5 h by suppressing SOCS1, while simultaneously increasing SOCS3 expression. Cm (MOI of 2) likewise induced the differential expression of SOCS1 and SOCS3, albeit less, and IL-10 dose-dependently regulated their expression, particularly SOCS3 (Figure 2(c)).

Next, experiments were conducted to ascertain whether SOCS1 and SOCS3 expressions are dose-dependently induced by chlamydial stimulants and if the observed differential regulation of SOCS by IL-10 is dependent on the stimulant concentration. We show in Figure 2(d) that rMOMP dose-dependently upregulated SOCS1 and SOCS3, with again a SOCS3 expression. With added IL-10, SOCS1 was reduced, and SOCS3 enhanced; likewise, IL-10 regulation of SOCS1 and SOCS3 was directly proportional to the stimulant concentration. Similarly, Cm at all MOIs stimulated SOCS1 and SOCS3 gene transcripts, especially SOCS3, and the addition of IL-10 to increasing MOIs of Cm did not alter SOCS1 but significantly (*P* <0.001) upregulated SOCS3 expression (Figure 2(e)). These findings confirm the differential regulation of chlamydial-induced SOCS1 and SOCS3 by IL-10, irrespective of the stimulant concentration.

To ensure the IL-10 differential regulation of SOCS, we calculated their SOCS1 and SOCS3 ratios, which are good indicators of the magnitude of their expression. As indicated in Figure 2(f), IL-10 reduced the SOCS1/SOCS3 ratios in rMOMP- and Cm-stimulated macrophages by ~4- and 7-fold, respectively, due to its down-regulation of SOCS1. Conversely, IL-10 enhanced, respectively, the SOCS3/SOCS1 ratio by ~4- and 8-fold fold in rMOMP and Cm cultures apparently by increasing SOCS3 expression (Figure 2(g)). These experiments infer that SOCS1 and SOCS3 are differentially induced and regulated by IL-10, and are key protagonists in the IL-10- inhibition of *Chlamydia-*induced inflammatory responses in macrophages.

The functions of SOCS1 and SOCS3 were quantified at the translational level employing flow cytometry to broaden our understanding of their expression and regulation as induced by chlamydial stimulants alone or with added IL-10. Flow cytometric analyses revealed the expression of SOCS1 (Figure 2(h)), and SOCS3 (Figure 2(k)) in chlamydial- and IL-10-stimulated macrophages by their increased fluorescence intensities as compared to uninfected macrophages. Once more, IL-10 reduced SOCS1 (Figures 2(i) and 2(j)) and enhanced SOCS3 (Figures 2(l) and 2(m)) expression. Immunofluorescence microscopy was also employed to provide visual evidence for the expression and regulation of SOCS by IL-10 in chlamydial macrophages as supporting protein data. Only SOCS3 data is shown because of difficulties in attaining good images for SOCS1 due to their lower expression. In Figure 2(n), the rows, respectively, reflect overlay images (merge; bright yellow fluorescence), nuclei (DAPI; blue fluorescence), macrophage surface (actin; red fluorescence), and SOCS3 (yellow fluorescence). Our results demonstrate that unstimulated cells displayed negligible SOCS3 expression. Although IL-10 upregulated SOCS3, it was lesser in contrast to rMOMP and Cm macrophages that exhibited increased bright yellow fluorescence aggregating around the nuclei, confirming their ability to induce high levels of SOCS3, which were enhanced with added IL-10. This study further confirms the induction of SOCS3 by chlamydial stimulants and the ability of IL-10 to regulate its translational expression and hence functions in macrophages.

### 3.3. Effect of MAPK Pathway-Specific Inhibition on Inflammatory Mediators and SOCS1 and SOCS3 Expressions

**Figure 3:**
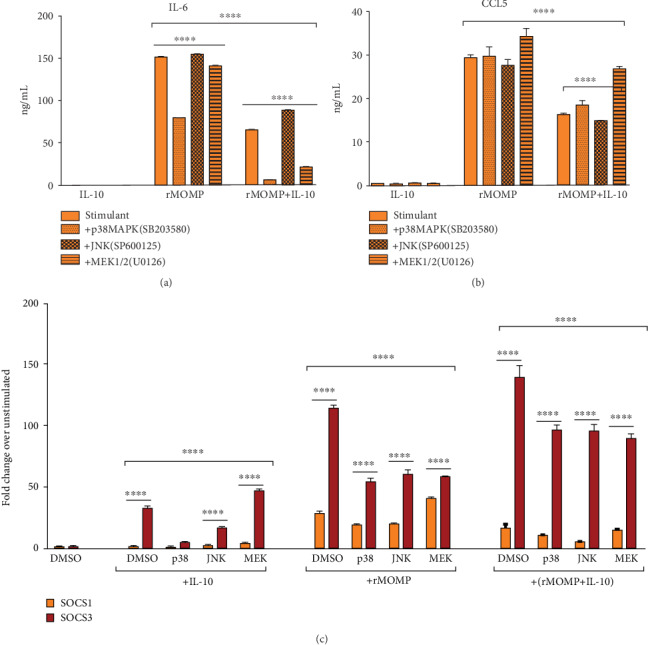
Specific MAPK pathways regulate the IL-10-inhibition of inflammatory mediators in chlamydial macrophages and the induction of SOCS expression. Macrophages (10^6^/mL) were pre-incubated for 1 h with MAPK pathway specific-inhibitors (each at 20 *μ*M) for p38 (SB203580), JNK (SP600125) and MEK1/2 (UO126) followed by stimulation for 24 h with rMOMP (10 *μ*g/mL) in the presence and absence of IL-10 (10 ng/mL). IL-6 (A) and CCL5 (B) were measured by specific ELISAs. TaqMan qRT-PCR was employed to quantify SOCS1 and SOCS3 mRNA gene transcripts (C). Results are presented as fold increase over the control. An asterisk indicates significant differences (*P* <0.05), and *P* values were calculated as described in Figure 1 legend. Each bar represents the mean ± SD of samples run in triplicates. Each experiment was repeated 3 times.

We investigated the downstream signaling pathways that may be involved in the induction and regulation of SOCS1 and SOCS3 in a *Chlamydia-*activating macrophage environment that may contain IL-10. We focused on MAPK pathways, specifically p38, JNK, and MEK1/2, that reportedly play pivotal roles in regulating immune-mediated inflammatory responses [59–61] and SOCS functions [62]. Macrophages were pre-incubated for 1 h with pathway-specific inhibitors targeting p38 (SB203580), JNK (SP600125) and MEK1/2 (U0126) before stimulation with rMOMP with and without added IL-10. Macrophages exposed to IL-10 secreted minimal or no IL-6 or CCL5 (Figures 3(a) and 3(b)). We show in Figure 3(a) that the p38 inhibitor significantly repressed (*P* <0.0001) IL-6 release, suggesting that p38 is necessary for IL-6 synthesis, corroborating reports that p38 inhibition reduces pro-inflammatory cytokine production [59, 63–66]. Inhibitors of the MEK1/2 and JNK pathways did not appreciably alter IL-6 expression and, as such, not required for its synthesis. Both p38 and MEK1/2 inhibitors enhanced the IL-10 anti-inflammatory effect, implying that they regulate the magnitude of IL-10 inhibition of inflammatory mediators. Contrastingly, the JNK inhibitor enhanced IL-6, which reveals its necessity for IL-10 modulation of IL-6. Both p38 and JNK inhibitors did not alter CCL5 expression, but noticeably CCL5 release increased with MEK1/2 inhibition in IL-10 co-cultures, suggesting that MEK1/2 blocks the IL-10 suppressive activity in macrophages (Figures 3(a) and 3(b)).

Next, quantification of SOCS1 and SOCS3 mRNA gene transcripts was conducted to determine the effect of pathway-specific inhibition on their expression, given the pivotal effects of specific pathways on cytokine and chemokine expression. Blockage of p38 caused the most notable decline in SOCS1 and SOCS3 mRNA expression, followed by JNK blockage, while blocking of MEK1/2 upregulated SOCS1 and SOCS3 expression as induced by IL-10. SOCS1 and SOCS3 transcripts were reduced by blocking p38 and JNK; conversely, MEK1/2 blockage enhanced SOCS1 but reduced SOCS3. Of interest, inhibiting p38, JNK, and MEK1/2 in co-cultures of rMOMP and IL-10 significantly reduced (*P* <0.001) SOCS1 and SOCS3 expressions (Figure 3(a)). Collectively, these results reveal that MAPK pathways regulate the induction of SOCS1 and SOCS3 in macrophages as triggered by either IL-10 and rMOMP or a combination of both.

### 3.4. Endogenous IL-10 Regulates Inflammatory Mediators and SOCS Expression in Chlamydial-Stimulated Macrophages

**Figure 4:**
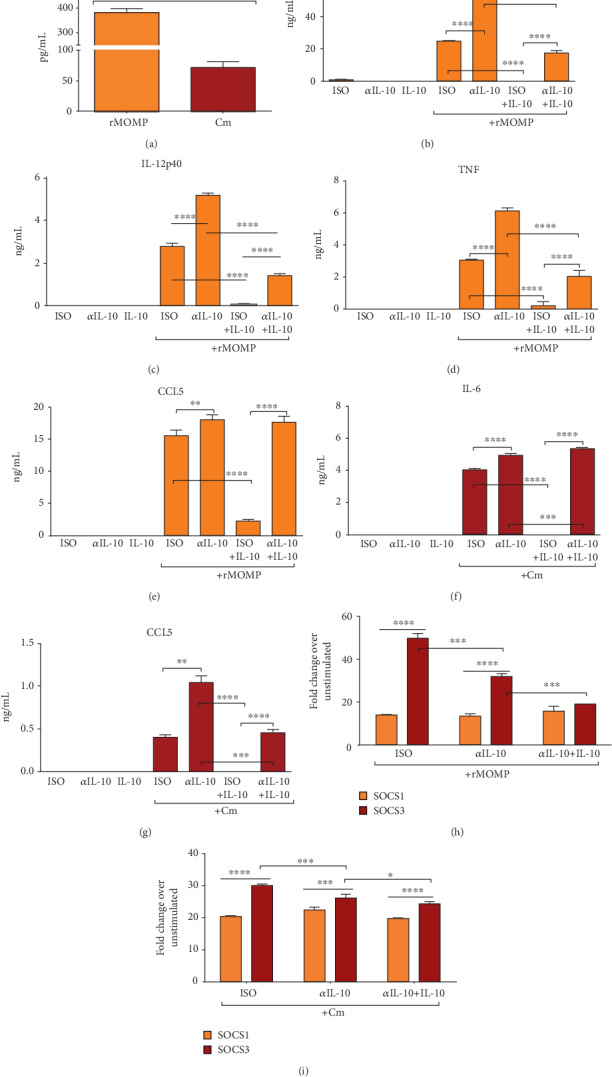
Endogenously produced IL-10 regulates inflammatory mediators and SOCS expression in chlamydial-stimulated macrophages. Macrophages (10^6^/mL) were stimulated for 24 h with rMOMP (10 *μ*g/mL) or Cm (MOI of 2) to quantify the production of endogenously produced IL-10 in supernatants by ELISA (A). Macrophages were pre-incubated with neutralizing Ab (*α*IL-10) to IL-10 (25 *μ*g/mL) for 30 min before adding rMOMP (10 *μ*g/mL) or Cm (MOI of 2) for an additional 24 h. Normal rat IgG1 Ab served as the isotype control (ISO). Post-stimulation, supernatants were collected to quantify IL-6 (B, F), IL-12p40 (C), TNF (D), and CCL5 (E, G) by specific ELISAs and RNA was isolated to quantify the mRNA gene transcripts of SOCS1 and SOCS3 (H-I) by TaqMan qRT-PCR. An asterisk indicates a significant difference (*P* <0.05), and *P* values were calculated as described in Figure 1 legend. Each bar represents the mean ± SD of samples run in triplicates, and each experiment was repeated at least 3 times.

Our data established that exogenous IL-10 effectively inhibited inflammatory mediators in macrophages exposed to chlamydial stimulants, and differentially regulated SOCS1 and SOCS3 expression. We questioned whether endogenously produced IL-10 might function similarly in chlamydial-stimulated macrophages. IL-10 secreted by rMOMP and Cm macrophages, respectively, were~381 pg/mL and~72 pg/mL (Figure 4(a)). Negative controls (isotype (ISO), anti-IL-10, and IL-10) did not induce any inflammatory mediators (Figures 4(b)–4(g)). Also, the anti-IL-10 Ab sufficiently neutralized the endogenously produced IL-10 in stimulated cultures, thus confirming its neutralization efficiency (Data not shown). Once more, rMOMP stimulated high levels of IL-6, IL-12p40, TNF and CCL5 and neutralization of the endogenously produced IL-10 resulted in the upregulation of these mediators, implying that endogenous IL-10 represses their maximal expression levels. Moreover, neutralization of exogenously added IL-10 with the anti-IL-10 Ab abolished the inhibitory effect of IL-10 in stimulated macrophages by restoring IL-6, IL-12-p40, TNF and CCL5 to comparable levels as induced by stimulants alone (Figures 4(b)–4(e)). These results were confirmed in Cm-exposed cultures whereby IL-6 and CCL5 were upregulated by neutralization of IL-10 (Figures 4(f)-4(g)). These findings reiterate that IL-10 (endogenous and exogenous) regulates the production of inflammatory mediators, thus solidifying the role of IL-10 in modulating inflammatory responses triggered by *Chlamydia* in macrophages.

To assess whether SOCS1 and SOCS3 mRNA gene transcripts would be altered by the removal of the endogenously produced IL-10, we quantified their expressions in chlamydial-stimulated macrophages. The removal of the endogenous IL-10 suppressed SOCS3 in both chlamydial cultures, proving that SOCS3 regulates the inflammatory responses. Co-culturing of the anti-IL-10 Ab with exogenous IL-10 lowered SOCS3 but not SOCS1 expression; thus confirming that exogenous and endogenous IL-10 effectively regulate SOCS expression (Figures 4(h)–4(i)). The reduced SOCS3 expression and its correlation with an increase in cytokines and chemokines support SOCS3 as a mediator of IL-10-inhibitory activity in a chlamydial macrophage environment and underscores SOCS3 in mediating the inhibitory effects of IL-10 and its IL-10-mediated immune responses [49, 67].

### 3.5. Effect of Exogenous IL-6 and TNF on Inducing and Regulating Inflammatory Mediators as Well as SOCS1 and SOCS3 in Chlamydial-Stimulated Macrophages

**Figure 5:**
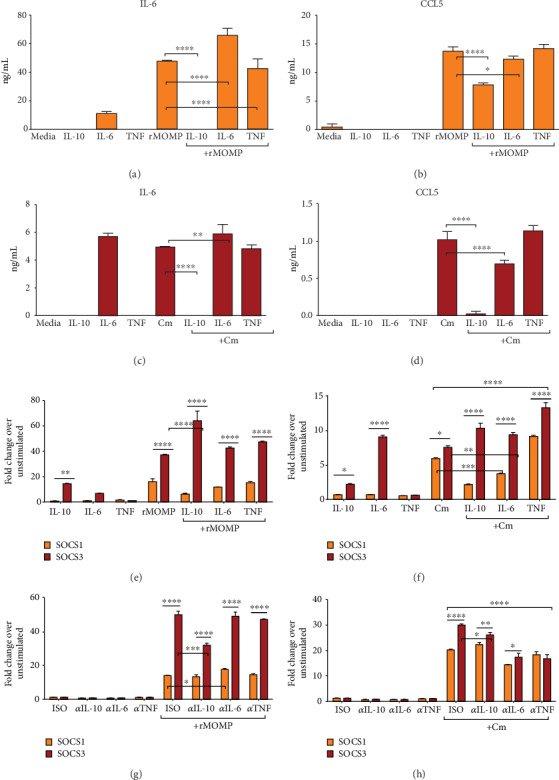
The effect of exogenous and endogenously produced IL-6 and TNF on the expression of inflammatory mediators and SOCS in chlamydial-stimulated macrophages. Macrophages (10^6^/mL) were stimulated with rMOMP (10 *μ*g/mL) or Cm (MOI of 2) in the presence and absence of IL-10, IL-6, and TNF (each at 10 ng/mL) for 24 h. RNA and cell-free supernatants were collected to quantify IL-6 and CCL5 (A-D) along with SOCS1 and SOCS3 mRNA gene transcripts (E-F), respectively, by TaqMan qRT-PCR and specific ELISAs. Macrophages were pre-incubated with neutralizing Abs to IL-10, IL-6, and TNF (each at 25 *μ*g/mL) for 30 min before adding rMOMP or Cm for an additional 24 h. Normal rat IgG1 Ab served as the isotype control (ISO). RNA was collected to quantify SOCS1 and SOCS3 mRNA gene transcripts (G-H). An asterisk indicates a significant difference (*P* <0.05), and *P* values were calculated as described in Figure 1. Each bar represents the mean ± SD of samples run in triplicates. Each experiment was repeated at least 3 times.

The endogenous study solidified the anti-inflammatory role of IL-10 and with SOCS1 and SOCS3 as the putative mediators of its inhibitory actions in chlamydial macrophage cultures. Because chlamydial stimulants can induce a plethora of inflammatory cytokines, we questioned whether other cytokines might contribute to SOCS induction in macrophages. We targeted IL-6 and TNF because they reportedly can stimulate SOCS1 and SOCS3 expression in macrophages [52]; and also to evaluate if they exhibit any anti-inflammatory properties. Macrophages were exposed to chlamydial stimulants with and without exogenously added IL-6, TNF and IL-10 to quantify IL-6 and CCL5. IL-10, as expected, decreased the expression of IL-6 and CCL5; IL-6 reduced CCL5, and TNF did not alter IL-6 or CCL5 production levels in chlamydial-stimulated macrophages (Figures 5(a)–5(d)). This data shows that IL-6 exhibits some anti-inflammatory effect, albeit to a lesser extent than IL-10.

We next investigated whether IL-6 and TNF contributed to SOCS1 and SOCS3 induction by chlamydial stimulants (Figures 5(e)–5(f)). Again, IL-10 alone or combined with stimulants significantly (*P* <0.0001) upregulated SOCS3, while simultaneously decreasing SOCS1 expression. Exogenous IL-6 induced more SOCS3 than SOCS1 expression; conversely, their induction by TNF was weak. The addition of IL-6 to chlamydial macrophages significantly (*P* <0.001 to 0.0001) reduced only SOCS1 expression. Contrastingly, TNF enhanced SOCS1 and SOCS3 expressions only in Cm and not rMOMP cultures, suggesting possibly the triggering by other chlamydial antigens. These findings indicate that IL-6 but not TNF can induce SOCS3; however, both cytokines regulate SOCS1 and SOCS3 expression. We speculate that perhaps, to resolve and balance the addition of these pro-inflammatory mediators to the chlamydial milieu, IL-6 and TNF may display transient anti-inflammatory tendencies, consistent with their pleiotropic properties [68].

Neutralization of endogenously produced IL-6 and TNF by their respective Abs did not significantly perturb rMOMP-induced SOCS1 and SOCS3 expression, implying their direct stimulation by rMOMP. Surprisingly, diminished SOCS1 and SOCS3 expressions occurred with blockade of endogenously produced IL-6 and TNF in Cm-exposed cultures, which corroborate reports that SOCS1 and SOCS3 provide significant negative feedback interchangeably for IL-6 and TNF [69]. The removal of endogenously produced IL-10 increased SOCS1 expression (Cm only), and reduced SOCS3, suggesting an essential role of IL-10 in the regulation of SOCS1 and SOCS3 expression (Figures 5(g)–5(h)). Collectively, our data sheds light on the potential roles of these cytokines implicated during chlamydial infections, but more importantly, how IL-10 regulates *Chlamydia* inflammation via SOCS proteins.

### 3.6. Chlamydial Stimulants Trigger an M1 Pro-Inflammatory Phenotype That Is Skewed to an M2 Anti-Inflammatory Phenotype in the Presence of Exogenous IL-10

**Figure 6:**
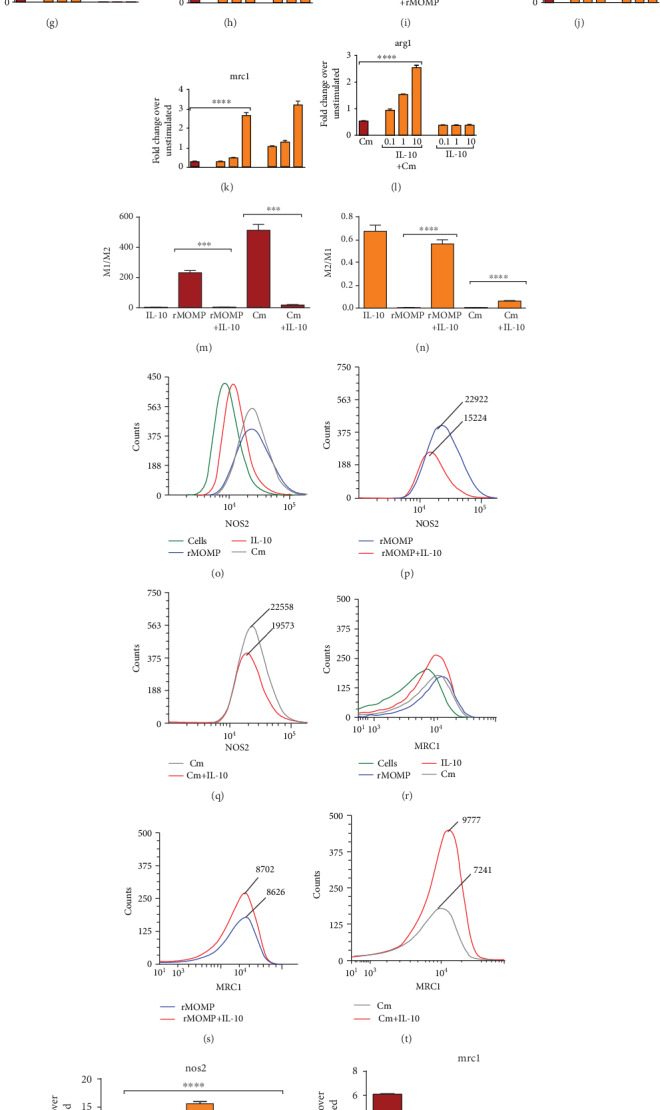
Skewing of the chlamydial M1 pro-inflammatory phenotype to an M2 anti-inflammatory phenotype by exogenous and endogenously produced IL-10. Macrophages (10^6^ cells/mL) were stimulated with rMOMP (0.1, 1 and 10 *μ*g/mL) (A-C) or infected with Cm (MOI of 0.5, 1 and 2) (D-F) in the presence and absence of IL-10 (10 ng/mL). Macrophages were stimulated with rMOMP (1 *μ*g/mL; G-I) or Cm (MOI of 2; J-L) in the presence and absence of IL-10 (0.1, 1, and 10 ng/mL). At 24 h post-stimulation, the mRNA transcripts of the m1 marker; nos2 and m2 markers; arg1 and mrc1 were quantified using TaqMan qRT-PCR. The m1/m2 (M) and m2/m1 (N) ratios were calculated from macrophages exposed to rMOMP or Cm in the presence and absence of IL-10. Protein expressions of nos2 and mrc1 were evaluated from stimulated cells employing flow cytometry (O-T). Macrophages pre-incubated with neutralizing Abs to IL-10, IL-6, and TNF were stimulated with rMOMP or Cm for an additional 24 h. Normal rat IgG1 Ab served as the isotype control (ISO). Post-stimulation, RNA was isolated to quantify the gene transcripts of nos2 and mrc1 (U-X) by TaqMan qRT-PCR. An asterisk indicates significant differences (*P* <0.05), and *P* values were calculated as described in Figure 1. Each bar represents the mean ± SD of samples run in triplicates, and each experiment was repeated at least 3 to 4 times with a representative experiment shown.

We have established above that IL-10 upregulates SOCS3 while simultaneously downregulating SOCS1, suggesting its capacity to stimulate a therapeutic M2 macrophage phenotype. Therefore, we phenotypically characterized chlamydial-stimulated macrophages (M1 and M2) to address if IL-10 polarizes macrophage phenotypes to exert its anti-inflammatory actions and to assess the role of SOCS1 and SOCS3 in skewing a chlamydial activating macrophage environment towards an M1 or M2 polarizing phenotype. Dose-dependent experiments were conducted using discriminate M1 (nos2) and M2 (arg1 and mrc1) markers to identify the macrophage phenotypic populations. Both M1 and M2 transcriptional expressions varied between experiments, but their expression patterns were consistent (Figures 6(a)–6(l)). Macrophages incubated with rMOMP or Cm at all tested concentrations expressed higher nos2 than those incubated with IL-10, implying the triggering of an M1 pro-inflammatory phenotype by chlamydial stimulants (Figures 6(a), 6(d), 6(g) and 6(j)). Interestingly, incubating IL-10 with stimulants significantly (*P* <0.001) reduced nos2 expression and skewing of the chlamydial M1 pro-inflammatory phenotype by IL-10. Nos2 expression increased with increasing concentrations of chlamydial stimulants, and IL-10 reduced nos2 expression at all tested dosages.

Our results for the M2 phenotypic markers conversely revealed that all concentrations of chlamydial stimulants induced minimal expression of mrc1 and arg1. In contrast, all dosages of IL-10 increased their expression, indicating selective stimulation of the M2 phenotype by IL-10 to possibly aid in mediating its anti-inflammatory actions (Figures 6(b)-6(c), 6(e)–6(f), 6(h)–6(i), 6(k)–6(l)). Co-incubation of IL-10 with chlamydial stimulants enhanced the expression of mrc1 and arg1 in macrophages, which infers that IL-10 skews chlamydial M1 towards an M2 phenotype to repress inflammation or that chlamydial stimulants skew M2 towards an M1 phenotype to balance macrophages polarizing functions in the microenvironment. We also calculated the M1 and M2 ratios as a predictive correlation of SOCS1 and SOCS3 expressions that regulate macrophage polarization. Depiction of the M1/M2 ratios indicates higher ratios for chlamydial stimulants, which are suggestive of enhanced M1 and SOCS3 expression, which were reduced with added IL-10 (Figure 6(m)). Contrastingly, higher M2/M1 ratios were attained for IL-10 and IL-10 co-incubated with stimulants, indicative of an IL-10 polarizing M2 phenotype (Figure 6(n)).

Flow cytometric analysis similarly demonstrated at the protein level that chlamydial stimulants triggered the high expression of nos2 with a concomitant lesser expression of mrc1. Once more, IL-10 reduced the expression of nos2 while simultaneously increasing that of mrc1, thus further illustrating the skewing of the chlamydial-induced M1 pro-inflammatory to an M2 anti-inflammatory phenotype at the translational level (Figures 6(o)–6(t)).

The observed skewing of the chlamydial M1 pro-inflammatory phenotype to an M2 anti-inflammatory macrophage phenotype by exogenous IL-10 begged to address the role of endogenously produced IL-10 in this phenomenon. As anticipated, IL-10 did not induce nos2 but rather upregulated mrc1 (Figures 6(u)–6(x)). Chlamydial stimulants in the presence of the isotype control Ab (ISO) upregulated nos2. But neutralization of the endogenously produced IL-10 significantly upregulated (*P* <0.0001) nos2, suggesting that the endogenously produced IL-10 regulates the M1 phenotype comparable to the exogenous IL-10 and its removal enhances the chlamydial M1 pro-inflammatory phenotype (Figures 6(u) and 6(w)). Contrastingly, chlamydial stimulants and ISO induced low mrc1 expression and removal of the endogenous IL-10 failed to upregulate mrc1 and skew chlamydial-stimulated macrophages to an M2 phenotype (Figures 6(v) and 6(x)). Addition of neutralizing Ab to stimulants with added IL-10 validates the significance of IL-10 in skewing the M1 phenotype (Figures 6(u)–6(x)). Our results provide a compelling role for IL-10 (endogenous and exogenous) in regulating chlamydial macrophage functions and exerting its anti-inflammatory effects.

### 3.7. Polarization of M1 and M2 Phenotypes at the Translational Level by Exogenous IL-10 in *Chlamydia*-Stimulated Macrophages

**Figure 7:**
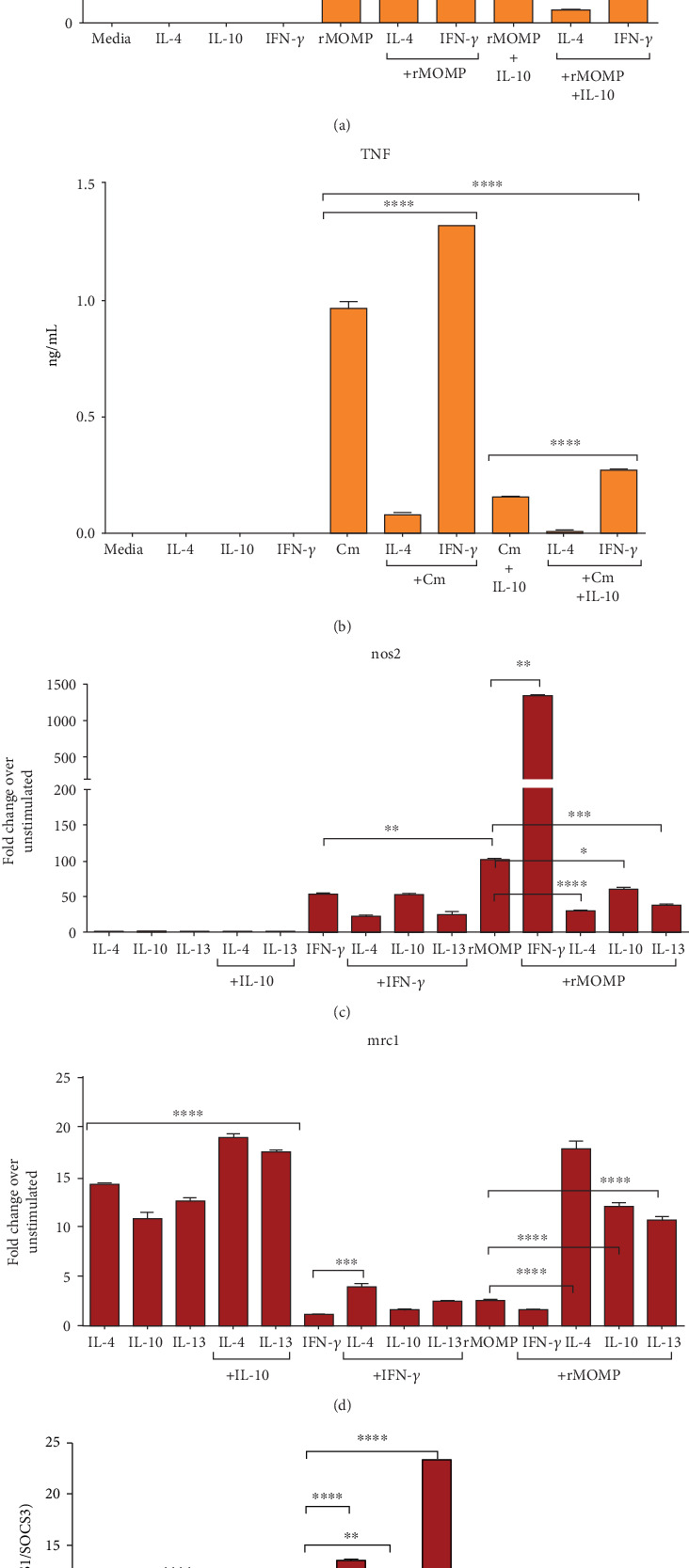
Impact of macrophage polarization on TNF expression, macrophage phenotypes as well as SOCS ratios in rMOMP cultures. Macrophages (10^6^/mL) were pre-incubated with IL-4 or IFN-*γ* (each at 10 ng/mL) for 1 h followed by stimulation with either Cm or its rMOMP in the presence and absence of IL-10 for an additional 24 h. Cell-free culture supernatants were collected to quantify TNF by ELISA (A-B). Macrophages were pre-incubated with IL-4, IL-10, IL-13, or IFN-*γ* for 1 h followed by stimulation with rMOMP for an additional 24 h. The mRNA gene transcripts for nos2, mrc1, socs1 and socs3 (C-E) were quantified via TaqMan qRT-PCR. An asterisk indicates significant differences (*P* <0.05), and *P* values were calculated as described in Figure 1 legend. Each bar represents the mean ± SD of samples run in triplicates, and each experiment was repeated at least 3 to 4 times with a representative experiment shown.

Because IL-10 stimulated an M2 phenotype, we conducted more studies to ensure the fate of chlamydial M1 phenotype in an established M1 and M2 polarizing microenvironment. Reportedly, M1 macrophages are induced by IFN-*γ* or PAMPs, and M2 macrophages by IL-4 and IL-13 [70]. For this purpose, macrophages were pre-incubated for 1 h with IFN-*γ* to express a pro-inflammatory M1 phenotype or with IL-4 or IL-13 for a more reparative M2 phenotype, followed by incubation with chlamydial stimulants with and without IL-10. Using TNF as an inflammation marker, we first demonstrated the effect of polarization on TNF induction by chlamydial stimulants. As shown in Figures 7(a)–7(b), polarized macrophages did not secrete TNF. Also, IFN-*γ* polarized macrophages did not alter TNF levels in rMOMP cultures but significantly enhanced (*P* <0.0001) that of Cm, suggesting stimulation by other chlamydial antigens. A reduction in TNF was seen when chlamydial stimulants and IL-10 were added to IFN-*γ* polarized macrophages, indicating the IL-10 anti-inflammatory actions. Conversely, IL-4 polarized macrophages significantly (*P* <0.0001) down-regulated TNF in chlamydial cultures and when combined with IL-10 for an enhanced additive anti-inflammatory effect.

Further, we quantified M1 and M2 phenotypes of polarized macrophages, including those polarized by IL-13 and IL-10 and only for rMOMP. As depicted in Figure 7(c), nos2 mRNA gene transcripts were not significantly induced by IL-4, IL-10, nor IL-13 polarized macrophages either alone or combined. Both IFN-*γ*-polarized macrophages and rMOMP alone stimulated marked expression of nos2 and when co-cultured, increased nos2 expressions by 14 to 28-fold, suggesting a synergistic enhancement of the M1 pro-inflammatory phenotypes. Noted are that polarized IL-10, IL-4 and IL-13 macrophages co-cultured with rMOMP reduced nos2 expression; but only the polarized IL-4 and IL-13 macrophages reduced nos2 expression as induced by IFN-*γ*.

Opposing results were attained for mrc1 where polarization of macrophages with IL-10, IL-4 and IL-13, but not IFN-*γ*, significantly increased (*P* <0.0001) expression of mrc1 alone or when IL-10 was delivered with IL-4 or IL-13, suggesting the selective stimulation of the M2 phenotype by these polarizing cytokines (Figure 7(d)). Only IL-4-polarized macrophages significantly skewed (*P* <0.001) the M1 phenotype of IFN-*γ* (up to 2-fold), suggesting an efficient IL-4 polarization. Contrastingly, polarized IL-4, IL-10, and IL-13 macrophages exposed to rMOMP significantly enhanced (*P* <0.0001) mrc1 expression (up to 5-fold), which indicates skewing of an M1 phenotype to an M2 phenotype. These results disclose and corroborate the skewing of the chlamydial pro-inflammatory M1 phenotype towards a repressive inflammatory M2 phenotype, not only by IL-10 polarization, but also by IL-4 and IL-13, especially IL-4 with its potent anti-inflammatory activity in stimulated macrophages. Moreover, the data highlights that polarization of macrophage functions and phenotypes by IL-10 is multifaceted and dependent on the stimulating microenvironment.

Analysis of the SOCS1/SOCS3 ratios of polarized macrophages revealed that the IL-10 SOCS1/SOCS3 ratio was low as compared to the IL-4 and IL-13 M2 phenotypes, further indicating differential induction of SOCS by these cytokines (Figure 7(e)). Delivery of IL-10 to polarized IL-4 or IL-13 macrophages decreased their SOCS1/SOCS3 ratios, implying that IL-10 regulates their M2 phenotypes. The rMOMP-induced SOCS1/SOCS3 ratio in contrast to that of FN-*γ* was lower, implying higher SOCS1 induction by IFN-*γ*. Notably, IL-4 and IL-13 polarization increased the IFN-*γ*-induced SOCS1/SOCS3 ratio, while the ratio was reduced by IL-10, suggesting that IL-10 differentially regulates SOCS expression compared to the other M2 polarizing cytokines. IL-10, IL-4, and IL-13 polarization reduced the SOCS1/SOCS3 ratio stimulated by rMOMP; conversely, IFN-*γ* polarized macrophages increased the ratio, which again is indicative of IFN-*γ* inducing a high SOCS1 expression [71]. Our results confirm the polarizing role of IL-10 similarly to IL-4 and IL-13 but with differential regulation of SOCS1 and SOCS3 by their respective M2 polarized phenotypes.

### 3.8. The Proteasome Inhibitor Bortezomib Alters the Anti-Inflammatory Effect of Exogenous IL-10 in Chlamydial-Stimulated Macrophages and Regulates the Expression SOCS and STATs

**Figure 8:**
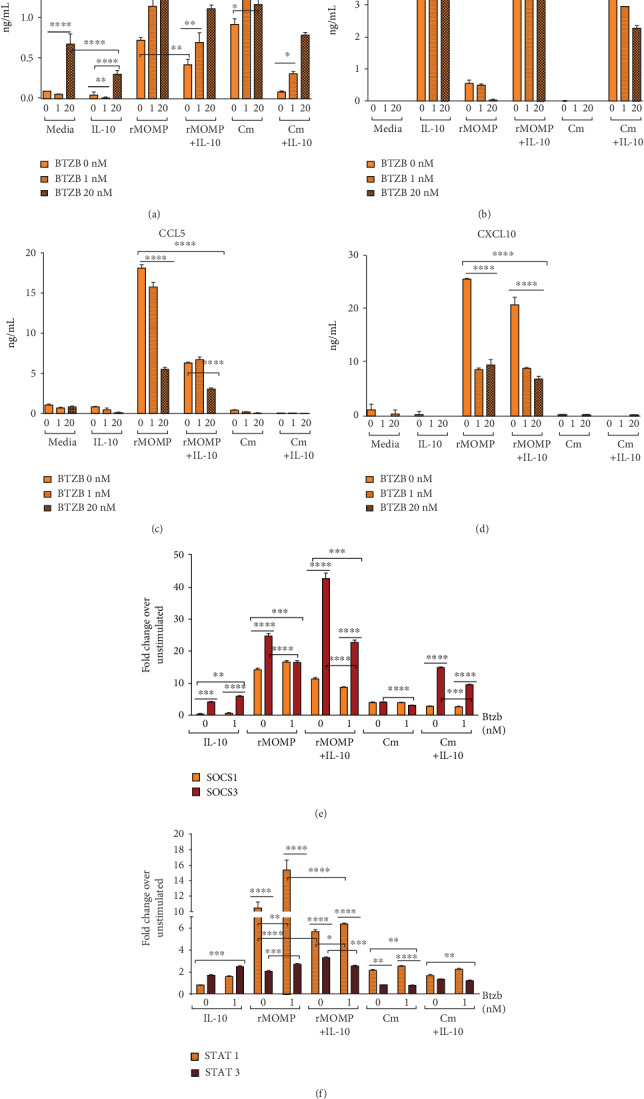
Inhibition of proteasomal degradation alters the IL-10 inhibition of inflammatory mediators and the transcriptional activation of SOCS and STATs in chlamydial macrophage cultures. Macrophages (10^6^/mL) were treated with 0, 1, and 20 nM of Bortezomib (Btzb) for 1 h, followed by stimulation with rMOMP (1 *μ*g/mL) or Cm (MOI of 2) in the presence and absence of IL-10 for 24 h. Post stimulation, cell-free culture supernatants were collected to quantify TNF (A), IL-10 (B), CCL5 (C) and CXCL10 (D) by specific ELISAs. The mRNA gene transcripts for socs1 and socs3 (E) and stat1 and stat3 (F) were quantified via TaqMan qRT-PCR. An asterisk indicates a significant difference (*P* <0.05), and *P* values were calculated as described in Figure 1 legend. Each bar represents the mean ± SD of samples run in triplicates. Each experiment was repeated at least 3 times.

It is well-established that SOCS1 and SOCS3 proteins mediate proteasomal degradation and ubiquitination through the ubiquitin-proteasome system, which is essential to controlling host innate and adaptive immune responses. We tested whether the IL-10-mediated down-regulation of inflammatory mediators is dependent on SOCS-mediated proteasome degradation by conducting experiments employing the proteasome inhibitor, Bortezomib (Btzb). Our results indicate that Btzb (at 20 nM) enhanced TNF levels in unstimulated and IL-10 cultures, suggesting blockade of TNF from proteasomal degradation (Figure 8(a)). Incubation of chlamydial stimulants with Btzb (at 1 and 20 nM) increased TNF production and reversed the IL-10-inhibition of TNF release in macrophages. In comparison, Btzb (at 20 nM) inhibited the endogenously produced IL-10 by rMOMP and with a negligible effect on an already low Cm-induced IL-10, a finding corroborating Btzb-inhibition of IL-10 production [72] (Figure 8(b)). Also observed is that Btzb suppressed the expression of CCL5 and CXCL10 in chlamydial macrophages, which were further suppressed by IL-10, suggesting that the IL-10-mediated inhibition of chemokines operates independently of proteasome degradation (Figures 8(c) and 8(d)). The results from this study are evidence to suggest that IL-10 inhibition of inflammatory mediators is multifaceted, and inhibition of proteasome degradation may be more efficient for the anti-inflammatory actions of IL-10 against cytokines than chemokines in chlamydial-stimulating macrophages.

To better understand the Btzb-mediated IL-10 regulation of inflammation, we elucidated the putative connections with SOCS1 and SOCS3 expression in macrophages. Btzb significantly enhanced (*P* <0.01) the IL-10-patterned reciprocal up-regulation of SOCS1 and SOCS3, which indicates that both are usual targets for proteasome degradation in an environment containing IL-10. Interestingly, in a chlamydial stimulating macrophage environment, Btzb increased SOCS1 (rMOMP only) and SOCS3 expression, correlating with its inhibition of the endogenously produced IL-10, and further illustrating SOCS1 and SOCS3 differential regulation by IL-10. Of utmost significance, Btzb exerted a significant inhibitory effect on SOCS1 (rMOMP only) and SOCS3 expression in chlamydial and IL-10 co-cultures, suggesting the Btzb-mediated IL-10 regulation of inflammation is mediated via the expression of SOCS1 and SOCS3 (Figure 8(e)).

Lastly, Figure 8(f) data discloses that IL-10, as expected, induced enhanced STAT3 than STAT1 expression, and both were significantly increased (*P* <0.001) after Btzb treatment. Not surprising, the chlamydial M1 macrophages exhibited increased STAT1 expression than STAT3, and both were enhanced by Btzb treatment. With added IL-10 to chlamydial cultures, STAT1 decreased with a corresponding increase in STAT3 expression, indicating their regulation by IL-10. However, with Btzb treatment of co-cultures, an upregulation of STAT1 with a concomitant decrease in STAT3 was observed, suggesting a Btzb dysregulation of STAT1 and STAT3. These results provide new revelations that the IL-10-mediated inhibition of cytokine and chemokines is mediated by SOCS1 and SOCS3 via proteasome degradation–dependent and independent pathways.

## 4. Discussion

*Chlamydia* immunopathology is responsible for the short- and long-term complications associated with the persistence of the disease due to its inflammatory nature. Many of the severe complications result from excessive immune responses at the site of infection; a tactic used by the bacterium to weaken host immune responses [6, 27, 73]. Effective antibiotics treatment is available, but the eradication of genital chlamydial diseases has been a challenge due to its asymptomatic nature [2, 26, 74]. Moreover, current therapeutic and preventive strategies that promote clearance of bacteria-mediated immune responses may also indirectly intensify pro-inflammatory responses, thereby exacerbating tissue damage [75, 76]. Su et al. and Batteiger et al. studies revealed that the accelerated eradication of genital *Chlamydia* by doxycycline also increased the population's susceptibility to reinfection by hindering the development of protective immunity [77, 78]. The asymptomatic nature of *Chlamydia* makes an identification, therapy, and prevention of sequelae a challenge [79]. As such, there is an urgency for an approved vaccine or alternative immune intervention strategy that can modulate inflammation while limiting pathology during the early infection. Previously, we reported that IL-10 is an efficacious regulator of *Chlamydia*-induced inflammatory responses in macrophages [27]. Here, several observations were made in deciphering the mechanisms of IL-10 inhibition of these inflammatory responses. These include 1) MOMP as a mediator of *Chlamydia* disease pathogenesis; 2) chlamydial stimulants triggered SOCS1 and SOCS3, but with more SOCS3 expression. IL-10 reciprocally regulated their expression by reducing SOCS1 and increasing SOCS3; 3) IL-10 (exogenous and endogenously produced) inhibited chlamydial inflammatory responses through the differential induction and regulation of SOCS1 and SOCS3 proteins; 4) the p38, JNK and MEK1/2 MAPK pathways are regulators of chlamydial inflammatory responses and SOCS1 and SOCS3; 5) chlamydial stimulants triggered an M1 pro-inflammatory phenotype that was skewed by IL-10 to an M2 anti-inflammatory phenotype to mediate its inhibitory actions, and 6) inhibition of proteasome degradation altered the expression of inflammatory mediators by suppressing SOCS1 and SOCS3, and dysregulating their STAT1 and STAT3 transcription factors.

Macrophages responsiveness to Pathogen-associated Molecular Patterns (PAMPs) can perpetuate inflammation, and *Chlamydia* proficiently infects and persists within macrophages, resulting in the recruitment of multiple innate immune effectors to the infection site [80–82]. Rajaram et al. demonstrated that macrophages play critical roles in innate and adaptive immunity against chlamydial infections [82]. It is well-established that macrophages serve as vehicles for the propagation and persistence of chlamydial elementary and reticulate bodies that are known to enhance its disease pathogenesis [83–87]. IL-10 anti-inflammatory effects on innate immune responses can further be attributed to macrophages and dendritic cells [88, 89]. Our previous study showed that live *Chlamydia* induced the secretion of pro-inflammatory mediators [26, 27], which corroborate reports of *Chlamydia* and its recombinant macrophage infectivity potentiator (rMip) stimulating the secretion of Interleukin-1*β* (IL-1*β*), TNF, IL-6, and IL-8 in infected human macrophages [90]. In the present study, rMOMP induced copious levels of inflammatory mediators, hence identifying MOMP as a mediator of chlamydial pathogenesis and its role in the establishment of early chlamydial infections. A study by Massari and colleagues delineated that MOMP proteosomes stimulated IL-6 and IL-8 secretions in endocervical epithelial cells [91]. Recently, Cheong et al. reported that MOMP triggered the release of pro-inflammatory cytokines in peripheral blood mononuclear cells (PBMCs) from *C. trachomatis*-infected patients [92]. The complexity of chlamydial diseases demands further investigations into the mechanisms by which chlamydial antigens activate host cell inflammatory responses at the site of colonization and infection for the disease pathogenesis, which are essential for proper insights in developing improved therapeutic strategies.

Herein, the potent IL-10 anti-inflammatory function [31, 93, 94] is now validated for chlamydial inflammation by impeding the release of not only cytokines but also chemokines in macrophages, which are congruent with our previous findings [27, 41]. Many investigators have reported on IL-10 inhibitory actions in macrophages exposed to protozoans (*Leishmania braziliensis* and *Leishmania chagasi*), viruses (Tacaribe Virus), fungal and bacteria (*Chlamydia* spp.*, Borrelia burgdorferi*, *Helicobacter pylori*, *Staphylococcus aureus*, *Streptococcus pneumoniae,* and *Yersinia enterocolitica*) [95]. Moreover, both human and murine T cells reactive to *Chlamydia* heat shock protein (Hsp60) produce IL-10 that can down-regulate Th1 immune responses [96]. Penttilä et al. demonstrated that while accelerated bacterial clearance was observed in IL-10 knock out (KO) mice, the absence of IL-10 inhibitory effect resulted in severe inflammation post *C. pneumoniae* infection [97], hence highlighting the importance of IL-10 in promoting host clearance of *Chlamydia* infections while also reducing pro-inflammatory responses. Reportedly, exogenously added IL-10 to infected C57BL/6 IL-10 KO mice down-regulated pro-inflammatory cytokines (IFN-*γ*, TNF, and IL-12) during an acute *C. pneumoniae* infection, which provides supporting evidence of an indispensable role of IL-10 in regulating *Chlamydia-*induced inflammation [98].

We further disclose that chlamydial simulants secrete IL-10 that self-regulates the magnitude of the concomitantly elicited inflammatory mediators. This observation indeed signals that *Chlamydia* might harness the immunosuppressive capacity of IL-10 to limit host initial anti-bacterial immune responses and facilitate its survival, or that reduction of excessive inflammatory mediators by the initial infection may be mediated via IL-10. Interestingly, Du et al. showed that *C. trachomatis* endogenously produced IL-10 modulated pro-inflammatory cytokines in human PBMCs [99]. This is consistent with a phenomenon where evolutionarily, pathogens can exploit the repressive function of IL-10 for their benefit to establish chronic infections [31, 88]. In this regard, Noto et al. reported that endogenous IL-10 promoted the optimal phagocytic activity of macrophages *in vitro*, and its administration to *Acinetobacter baumannii-*infected mice diminished a fatal outcome [100]. Others have documented that IL-10-deficient mice develop exacerbated fever in response to lipopolysaccharide (LPS) [101, 102] that also led to amplified secretion of IL-1*β*, IL-6, and TNF [103]. This highlights the importance of not only the role of IL-10 in immune regulation but also the impact of its dysregulation in many diseases [88, 102]. In contrast, reports have highlighted IL-10 to have different roles in infection models of the lymphocytic choriomeningitis virus [104], *Schistosoma mansoni* [105], *Mycobacterium tuberculosis* [106] and *Candida albicans* [107] where it impedes control and clearance of these pathogens [87]. While IL-10 may have opposing roles in several diseased situations, its function in chlamydial-induced inflammation holds promise for many therapeutic advantages. Overall, we delineate an encompassing role for IL-10 (endogenous and exogenous) in minimizing the excessive inflammatory responses induced by *Chlamydia* infections.

Our results reveal that chlamydial stimulants upregulated SOCS1 and SOCS3 in macrophages with higher SOCS3 expression, which underscore our previous findings [41]. Interestingly, endogenously produced IL-6 and TNF triggered by live Cm contributed to the overall expression of SOCS1 and SOCS3 since their abrogation reduced SOCS1 and SOCS3 expression. This unique observation for live Cm but not rMOMP may imply direct stimulation of SOCS1 and SOCS3 by rMOMP and probably de novo protein synthesis of SOCS by live Cm. The underlying consequence for the induction of SOCS1 and SOCS3 by *Chlamydia* may be a mechanistic ploy to limit its inflammation during early infection for perpetuation and survival in the host. It is well-known that SOCS1 and SOCS3 are rapidly induced by TLR agonists [108, 109], which in part explain their induction by a variety of bacterial pathogens, including *Borrelia burgdorferi* [39], *Listeria monocytogenes* [110], *Mycobacterium bovis* [111], *M. tuberculosis* [112], *Salmonella enterica* [113], and *S. aureus* [114], or bacterium-derived substances such as LPS [115, 116] and CpG-DNA [117]. Our previous [41] and current findings demonstrate that *Chlamydia* and its MOMP can differentially induce SOCS1 and SOCS3 in macrophages.

The results here elegantly unveiled that IL-10 immunomodulation of chlamydial inflammatory mediators in macrophages is mediated by simultaneously up-regulating SOCS3 and down-regulating SOCS1; thereby promoting SOCS proteins integral role in its anti-inflammatory actions. In this regard, Dennis et al. reported that co-stimulation of J774 macrophages with live spirochetes of *B. burgdorferi* and IL-10 resulted in IL-10 decreasing cytokines expression while enhancing SOCS1 and SOCS3 expression [39], further inferring that SOCS proteins are involved in the IL-10 anti-inflammatory effect in macrophages. Of significance, in the current study, neutralization of endogenously produced IL-10 resulted in a significant decrease in SOCS3 expression. The marked increase in inflammatory mediators and the reduced SOCS3 expression due to blockade of endogenous IL-10 supports SOCS3 as a mediator and regulator of IL-10-inhibitory activity in a chlamydial stimulating macrophage environment. This finding suggests that the removal of endogenous IL-10 while it may not impair total SOCS induction, it is essential for reparation of inflammatory responses.

Triggering of MAPK pathways leads to activation of the transcription factor, NFкB, with subsequent downstream induction of a plethora of inflammatory mediators [118], which are the hallmarks of many inflammatory diseases including *Chlamydia* [19, 26, 27]. Several of these pathways, including p38, MEK1/2, and JNK, regulated IL-10 inhibition of inflammatory mediators as well as the expression of SOCS1 and SOCS3 in our study. Abrogation of these pathways perturbed the immunosuppressive nature of IL-10, as denoted by their regulation of cytokine and chemokine expression. Guimarães et al. demonstrated that the anti-inflammatory compound, curcumin inhibited LPS-induced inflammatory cytokines in macrophages via mechanisms that involve modulating the expression and activity of SOCS1, SOCS3, and p38, which underscores our findings [119]. Inhibition of MAPK pathways has been exploited as potential therapeutics for diseases, including Alzheimer's [120] and inflammatory bowel disease [121]. Additionally, preclinical data targeting inhibitors of the p38 and JNK pathways suggest that they exhibit anti-inflammatory effects [122]. Consequently, a better understanding of signaling events will provide insight into the molecular mechanisms of IL-10-mediated reduction of inflammatory responses during an early *Chlamydia* infection. Given that blocking MAPK signaling pathways offered differential manifestations by both *Chlamydia* and IL-10, further understanding of tissue- and disease-specific regulatory mechanisms for MAPK signaling pathways might provide clues for the development of efficacious anti-chlamydial therapeutics.

Macrophages play an indispensable, anti-pathogenic role in the regulation and resolution of inflammation [123, 124]. An immunological paradigm exists where macrophages can be activated via an M1 and M2 dichotomy to maintain homeostasis within the cell environment [125, 126]. The M1 macrophage phenotype is characterized by the production of high levels of pro-inflammatory responses, whereas M2 macrophages are characterized by their anti-inflammatory activities [127]. Here we provide some evidence for the IL-10-dependent regulation of *Chlamydia*-induced inflammatory responses in both M1- and M2-activated and M1- and M2-polarized macrophages. *Chlamydia* stimulants upregulated the nos2, M1 marker, while barely inducing the arg1 and mrc1, M2 markers, coinciding with their pro-inflammatory potent M1 phenotypic activity. Our results are congruent with those of Tang et al., who showed that *M. tuberculosis* triggered macrophage polarization towards an M1 phenotype by producing high concentrations of inflammatory mediators, and also increasing M1 cells and decreasing M2 cells in infected patients [128]. Here, IL-10 downregulated nos2 genes and upregulated both arg1 mrc1 as induced by *Chlamydia* stimulants, suggesting its ability to skew macrophage phenotypes from an M1-like environment towards a more healing M2-like environment for its inhibitory actions.

Recent studies have established the potential for SOCS proteins to regulate M1 and M2 macrophage polarization [109]. Supposedly, a high SOCS1 to SOCS3 ratio could be a potential marker for M2 macrophages, while high SOCS3 to SOCS1 ratio is associated with M1 cells in *in vivo* experiments [109]. Contrastingly, we show that SOCS3, but not SOCS1, is efficiently up-regulated in M2 macrophages, and this rapid increase in SOCS3 may have a significant role in sustaining some features of the anti-inflammatory phenotype. For chlamydial M1 macrophages, SOCS1 is an essential regulator not only of pro-inflammatory mediators but also of IL-10 anti-inflammatory effects and, consequently, may act to prevent overshooting of the inflammatory response. Therefore, we speculate that modulation of SOCS1 expression represents a potential strategy to control imbalanced macrophage activation in inflammatory diseases. Reportedly, both M1-like and M2-like macrophages can be reprogrammed depending on the appropriate stimuli [125, 126]. In this regard, we found that the M2 activators, IL-4, and IL-10 synergistically decreased nos2 in chlamydial cultures while enhancing mrc1, highlighting the potential of these potent therapeutic cytokines in inflammatory response resolution. Our findings suggest that individual or combinations of M2 activators can efficiently reverse M1-like phenotypes towards a more M2-like environment to suppress chlamydial acute inflammatory responses. Moreover, because of the impact of M1/M2 activation in moderating host immune responses, identification, and modulation of macrophage phenotypes in inflammatory diseases seem therapeutically useful [129, 130].

The threshold, magnitude, and specific responses elicited by cytokine stimulation are regulated by numerous mechanisms, including SOCS proteins-mediated proteasomal degradation [131]. In the present study, Btzb increased TNF but suppressed CCL5 and CXCL10 secretions in chlamydial macrophages, suggesting an inverse relationship between blocking proteasomal degradation of cytokines versus chemokines, and its immunostimulatory and immunosuppressive effects. Our results are congruent with those of Cleophas et al., who demonstrated that Btzb reverses cytokine suppression by the drug romidepsin by increasing IL-1*β* in a patient gout model [132]. Likewise, reports by Sanacora et al. disclose that Btzb treatment increased IL-8 secretion in macrophages and monocytes [133], which supports the notion of additional cytokines as potential targets for proteasome degradation. Other investigators have shown that Btzb treatment down-regulated CXCL9 released by activated T cells [134], further underscoring our CCL5 and CXCL10 data.

Herein, Btzb treatment of chlamydial macrophages resulted in lower IL-10 secretions and altered IL-10 regulation of SOCS1 and SOCS3, which may account for IL-10 differential anti-inflammatory effects in regulating inflammatory mediators. Btzb suppressed SOCS1 and SOCS3 expression, especially SOCS3 in chlamydial macrophages both alone and co-incubated with IL-10, indicating that perhaps SOCS3 is more susceptible to proteasomal degradation than SOCS1. Noteworthy, our results disclosed that the decreased SOCS expression may be linked to the repression of IL-10 anti-inflammatory activity evidently by the restored TNF release. Interestingly, being that SOCS1 and SOCS3 are negative feedback regulators of STAT1 and STAT3, respectively, we further illustrate that Btzb treatment caused dysregulation of their expression in chlamydial cultures. These observations suggest that blocking of SOCS1 and SOCS3 actions by Btzb impacts STAT activity, which inadvertently influences the immunomodulatory functions of IL-10 in chlamydial inflammation. While there is limited information on the effects of Btzb on SOCS and STAT induction in the progression of chlamydial diseases, our result holds promise for a better understanding of the complexities of SOCS and other inflammatory pathways as utilized by *Chlamydia* and IL-10 for their opposing effects during an early *Chlamydia* infection and necessitate further investigations.

In conclusion, our study provides novel insights into the complex role of IL-10 in suppressing chlamydial inflammatory responses and identifies MOMP as a mediator of its disease pathogenesis. Our findings confirm that IL-10 inhibits chlamydial inflammatory responses in macrophages by activating p38-, JNK- and MEK1/2-MAPK dependent pathways coupled with simultaneously skewing chlamydial M1 pro-inflammatory towards a more reparative M2 phenotype and underscore SOCS1 and SOCS3 as mediators for its inhibitory actions. This data has important therapeutic implications in targeting IL-10 and SOCS in macrophages and, therefore, could be beneficial for controlling *Chlamydia* and other bacterial inflammatory diseases. Notably, the reciprocal regulation of SOCS1 and SOCS3 in macrophage by PLA-PEG-encapsulated IL-10 to prolong its biological half-life warrants more studies [41]. Our future research encompasses studying the *in vivo* role of SOCS1 and SOCS3 in the IL-10-mediated inhibition of chlamydial-induced genital inflammation to expound on the roles of these regulators in the remediation of *Chlamydia* inflammatory etiologies.

## Data Availability

The data used to support the findings of this study are available from the corresponding author upon request.
